# A *Drosophila* Smyd4 Homologue Is a Muscle-Specific Transcriptional Modulator Involved in Development

**DOI:** 10.1371/journal.pone.0003008

**Published:** 2008-08-20

**Authors:** Elizabeth C. Thompson, Andrew A. Travers

**Affiliations:** Cell Biology, MRC Laboratory of Molecular Biology, Cambridge, United Kingdom; Yale University, United States of America

## Abstract

**Background:**

SET and MYND domain (Smyd) proteins are involved in the transcriptional regulation of cellular proliferation and development in vertebrates. However, the *in vivo* functions and mechanisms by which these proteins act are poorly understood.

**Methodology/Principal Findings:**

We have used biochemical and genetic approaches to study the role of a Smyd protein in *Drosophila*. We identified eleven *Drosophila* genes that encode Smyd proteins. *CG14122* encodes a Smyd4 homologue that we have named dSmyd4. dSmyd4 repressed transcription and recruited class I histone deacetylases (HDACs). A region of dSmyd4 including the MYND domain interacted directly with ∼150 amino acids at the N-termini of dHDAC1 and dHDAC3. dSmyd4 interacts selectively with Ebi, a component of the dHDAC3/SMRTER co-repressor complex. During embryogenesis dSmyd4 was expressed throughout the mesoderm, with highest levels in the somatic musculature. Muscle-specific RNAi against dSmyd4 resulted in depletion of the protein and lead to severe lethality. Eclosion is the final moulting stage of *Drosophila* development when adult flies escape from the pupal case. 80% of dSmyd4 knockdown flies were not able to eclose, resulting in late pupal lethality. However, many aspects of eclosion were still able to occur normally, indicating that dSmyd4 is likely to be involved in the development or function of adult muscle.

**Conclusions/Significance:**

Repression of transcription by dSmyd4 and the involvement of this protein in development suggests that aspects of Smyd protein function are conserved between vertebrates and invertebrates.

## Introduction

Development requires the establishment and maintenance of patterns of gene expression. The activity of a gene is dependent on both the available repertoire of transcription factors and the local packaging of DNA into chromatin. Proteins involved in regulation of chromatin structure can therefore act as important determinants of developmental processes. SET and MYND domain (Smyd) proteins are conserved from yeast to vertebrates and the human and mouse genomes each contain five annotated Smyd proteins. SET domains were first identified in the *Drosophila* proteins Su(var)3–9, Enhancer of Zeste and Trithorax [1], [2], [3]. SET domains catalyse histone methylation [3], [4]. SET proteins are involved in both transcription regulation at specific loci and more widespread events such as heterochromatin formation [5]. The SET domains of vertebrate Smyd1, Smyd2 and Smyd3 catalyse methylation of H3-K4 [6], [7], [8]. In addition Smyd2 methylates H3-K36 and the non-histone substrate p53 [9], [10]. MYND (Myeloid translocation protein, Nervy, Deaf) domains are composed of two zinc fingers that mediate protein-protein interactions [11]. These domains are found in many proteins that regulate transcription, but their specific functions in Smyd proteins have not been determined. In other proteins MYND domains are involved in the recruitment of histone deacetylase (HDAC) containing complexes [11], [12], [13], [14]. HDAC complexes are conserved between eukaryotes and these complexes are recruited as co-repressors to remodel local chromatin structure [15].

Despite the prevalence of Smyd proteins throughout evolution their *in vivo* functions are poorly understood. Recent studies have implicated Smyd proteins in the transcriptional regulation of cellular proliferation and differentiation processes [7], [16]. Smyd3 is over-expressed in most hepatocellular and colorectal carcinomas and a number of gene targets have been identified [7]. Smyd1 is expressed specifically in muscle tissue and loss-of-function studies in vertebrates identified an important role for Smyd1 in muscle development [8], [16], but it is unclear how it fulfils this role *in vivo*. *Drosophila* provides a highly tractable system for *in vivo* studies of novel genes. We have identified eleven genes encoding Smyd homologues in the *Drosophila* genome. Data from gene expression databases indicate that six of these genes are expressed predominantly in the mesoderm, which develops to become muscle. Mesoderm is specified early in *Drosophila* embryogenesis [17]. Patterns of gene expression established within the mesoderm define regions of cardiac, visceral and somatic muscle [18], [19]. The somatic musculature is formed by the fusion and migration of groups of cells to form a stereotypical pattern of larval musculature [20], [21]. During the pupal stage these larval muscles are broken down and adult muscles are formed from pools of adult myoblasts specified during embryogenesis [22].

This study provides the first characterisation of a *Drosophila* Smyd protein. CG14122 (FlyBase accession number: FBgn0036282), named *Drosophila* Smyd4 (dSmyd4) is homologous to human Smyd4. Like vertebrate Smyd1 and Smyd2, *Drosophila* Smyd4 is able to repress transcription. dSmyd4 interacts directly with *Drosophila* class I HDACs via their N-termini. dSmyd4 is expressed throughout the mesoderm of *Drosophila* embryos and knockdown of dSmyd4 by RNAi results in lethality, predominantly at the late pupal stage. dSmyd4 loss-of-function results in a defect in eclosion of adult flies from the pupal case, suggesting an important role for dSmyd4 in development.

## Results

### Identification of *Drosophila* Smyd proteins

We performed basic local alignment search tool (BLAST) searches using each of the human Smyd homologues to comprehensively identify putative Smyd proteins encoded in the *Drosophila* genome. We identified eleven genes that contained both SET and MYND domains. Other BLAST hits were discarded. CG11160, CG12119, CG14122, CG14590, CG1868, CG18136, CG7759, CG8378, CG8503, CG9642 and MSTA contained well-conserved MYND and SET domains compared to human Smyd homologues (Fig S1). Gene expression data from FlyAtlas and the Berkeley *Drosophila* Genome Project *in situ* project suggest that most *Drosophila* Smyd proteins are expressed specifically in muscle, brain or sex specific tissues (Table S1) Supplementary Table 1; [23], [24]. The subcellular localisation of six of these proteins was analysed. CG14122, CG1868, CG11160 and CG8378 exhibited predominantly cytoplasmic over-expression patterns, whilst CG12119 was predominantly nuclear and CG8503 was exclusively cytoplasmic (Fig S2).

### CG14122 is homologous to human Smyd4

**Figure 1:**
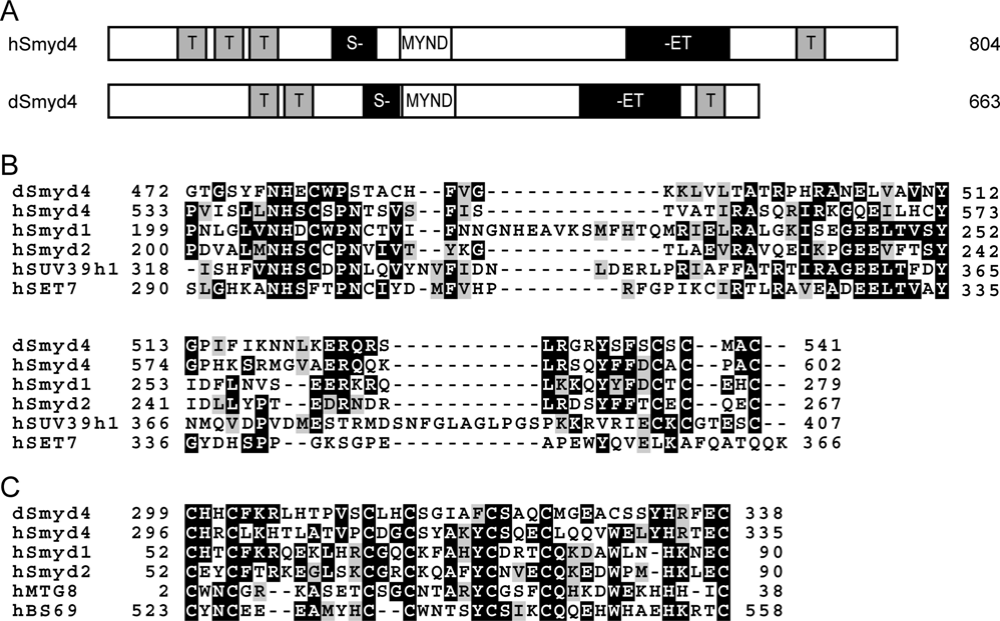
CG14122 is homologous to human Smyd4. A, Domain structure of human Smyd4 (hSmyd4) and *Drosophila* CG14122 (dSmyd4). T, tetratricopeptide repeat motif; S-, first part of the SET domain; MYND, MYND zinc fingers; -ET, second part of the SET domain, including cysteine-rich post-SET region. B, Alignment of the SET domains of dSmyd4 and human Smyd4, Smyd1, Smyd2, SUV39h1 and SET7. C, Alignment of the MYND domains of dSmyd4 and human Smyd4, Smyd1, Smyd2, MTG8 and BS69. In B and C identical residues are shaded black and similar residues are shaded grey.

The domain structures of CG14122 and human Smyd4 are shown in figure 1A. CG14122 shares 34% and 40% identity with the SET and MYND domains of human Smyd4 respectively (Fig 1B and C). CG14122 contains a split SET domain common to Smyd proteins. CG14122 also contains tetratricopeptide repeat motifs that are a feature of human Smyd4, but not other human Smyd proteins. We have named this protein *Drosophila* Smyd4 (dSmyd4).

### dSmyd4 directly recruits histone deacetylases

**Figure 2:**
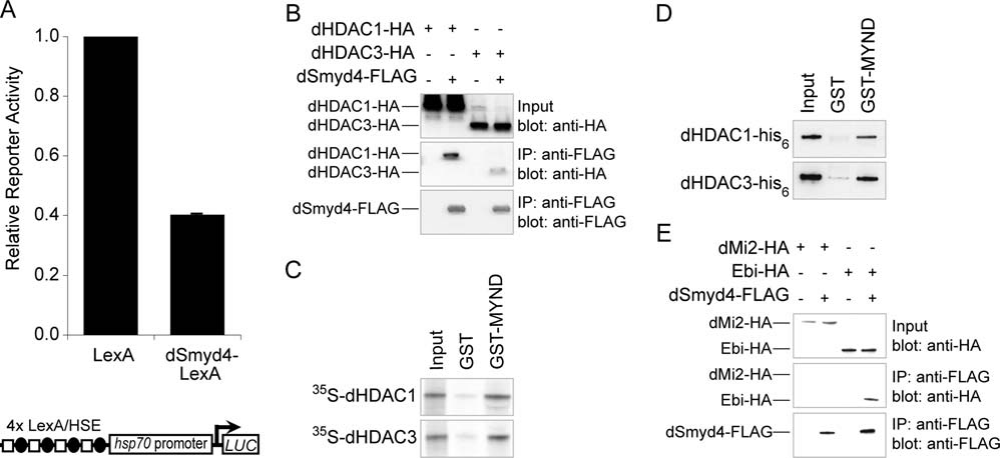
dSmyd4 represses transcription and interacts with HDACs. A, Activity of dSmyd4-LexA in a LexA dependent reporter assay. The reporter activity of LexA alone was normalised to a value of one. Results are the mean of three separate transfections. A schematic of the LexA dependent promoter is shown below. LexA, LexA binding sites; HSE, *Drosophila* heat shock elements; *hsp70* promoter, minimal *hsp70* promoter including TATA box; *LUC*, firefly luciferase gene. B, dHDAC1-HA and dHDAC3-HA co-immunoprecipitate with dSmyd4-FLAG. Input represents 10% of lysate used. C, A GST fusion of amino acids 208–377 of dSmyd4 (GST-MYND) or GST alone were used in pulldown assays with *in vitro* translated radiolabelled dHDAC1 or dHDAC3. Input represents 5% of *in vitro* translation reaction used. D, GST-MYND or GST alone were used in pulldown assays with recombinant dHDAC1-his_6_ or dHDAC3-his_6_. Input represents 2% of recombinant protein used. E, Ebi-HA, but not dMi2-HA, co-immunoprecipitates with dSmyd4-FLAG. Input represents 10% of lysate used.

**Figure 3:**
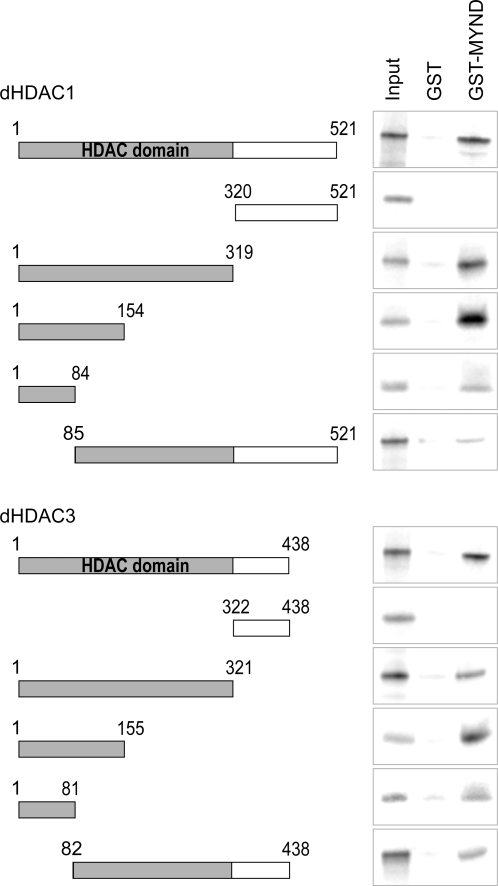
Mapping the interaction between dSmyd4 and dHDAC1 and dHDAC3. A GST fusion of amino acids 208–377 of dSmyd4 (GST-MYND) or GST alone were used in a pulldown assay with *in vitro* translated radiolabelled fragments of dHDAC1 or dHDAC3. The schematics on the left indicate the fragments used.

Since Smyd proteins are able to modulate transcription, we analysed the activity of a dSmyd4-LexA fusion at a LexA dependent promoter (Fig 2A). In a reporter assay in S2 cells dSmyd4 consistently repressed transcription between two and four-fold compared to LexA alone. Vertebrate Smyd1 and Smyd2 repress transcription by recruiting HDACs, therefore we tested whether the mechanism of repression is conserved [9], [16]. Both *Drosophila* class I HDACs, dHDAC1 and dHDAC3, co-immunoprecipitated with dSmyd4 (Fig 2B). The MYND domain containing protein MTG8 is implicated in the recruitment of HDAC co-repressor complexes [11], [13], [14]. We tested whether a fragment of dSmyd4 including the MYND domain (amino acids 208–377) was able to bind directly to dHDAC1 and dHDAC3. In pulldown assays GST-MYND interacted with both *in vitro* translated dHDAC1 and dHDAC3 (Fig 2C) and recombinant his_6_-tagged dHDAC1 and dHDAC3 produced in bacteria (Fig 2D). Although MYND domains are known to recruit HDAC containing complexes, a direct interaction between a MYND domain and HDAC has not previously been demonstrated. To define this interaction at higher resolution we used pulldown assays to map the region of interaction (Fig 3). ∼150 amino acids corresponding to the highly conserved N-termini of dHDAC1 and dHDAC3 were sufficient to bind dSmyd4 GST-MYND. Fragments of dHDAC1 and dHDAC3 lacking the first ∼80 amino acids exhibited only weak binding capacity.

### dSmyd4 interacts with Ebi

dHDAC1 and dHDAC3 exist in four independent co-repressor complexes in *Drosophila.* dHDAC1 is found in the NuRD, Sin3A and CoREST complexes, whereas dHDAC3 is a component of SMRTER, the *Drosophila* homologue of NCoR/SMRT complex [15]. We tested whether dSmyd4 co-immunoprecipitated with additional components of these complexes. dSmyd4 interacted with Ebi, a component of the dHDAC3/SMRTER complex [25], but not dMi2 a component of the NuRD complex (Fig 2E), or Sin3A or CoREST (data not shown). This indicates that dSmyd4 can be specifically engaged with the SMRTER co-repressor complex rather than participating in general interactions with all *Drosophila* HDAC complexes.

### dSmyd4 is expressed in the mesoderm

**Figure 4:**
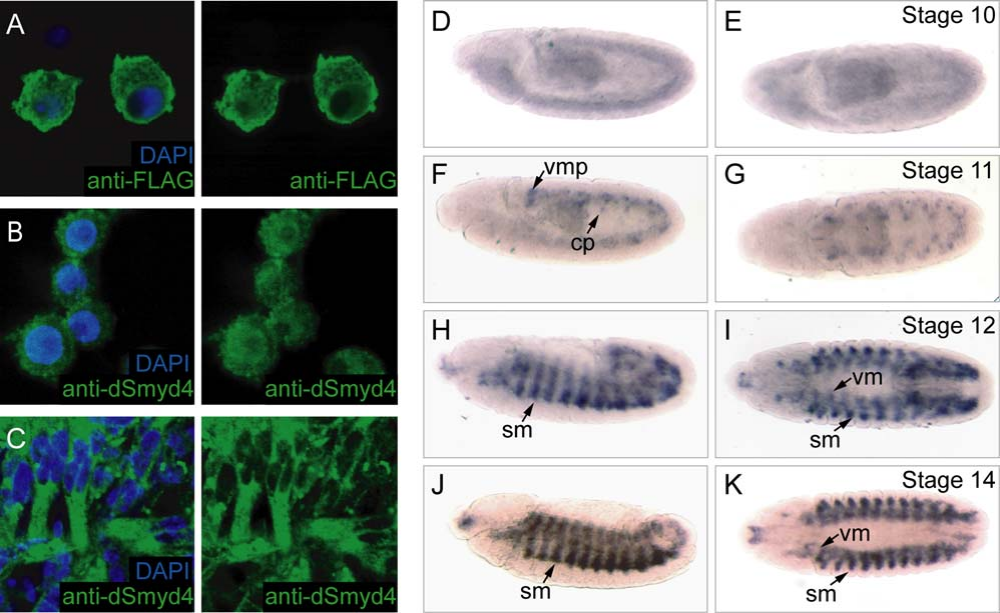
dSmyd4 is expressed in the embryonic mesoderm. A, dSmyd4-FLAG was over-expressed in S2 cells and visualised by immunofluorescence using anti-FLAG. B, Endogenous dSmyd4 in S2 cells was visualised by immunofluorescence using anti-dSmyd4. C, Endogenous dSmyd4 in late stage *Drosophila* embryos was visualised by immunofluorescence using anti-dSmyd4. In A, B and C, nuclei were visualised with 4′,6-diamidino-2-phenylindole (DAPI). D–K, *In situ* hybridisation of *dSmyd4* mRNA in developing *Drosophila* embryos, anterior to the left. D, F, H and J are lateral views; E, G, I and K are dorsal views. D and E, stage 10 embryo, weak expression of dSmyd4 throughout the mesoderm. F and G, stage 11 embryo, dSmyd4 expression in specified mesoderm. vmp, visceral muscle precursor; cp, cardiac precursor. H and I, stage 12 embryo, dSmyd4 was expressed at high levels in the somatic muscle (sm) and expression was maintained in visceral muscle (vm). J and K, stage 14 embryo.

To gain insight into the *in vivo* function of dSmyd4 we determined its expression pattern. Over-expressed dSmyd4 was predominantly cytoplasmic in S2 cells (Fig 4A). We generated an antibody against dSmyd4 that specifically recognised dSmyd4 in western blots and immunofluorescence (Fig S3). In S2 cells endogenous dSmyd4 showed a nuclear preference (Fig 4B). However, in late stage *Drosophila* embryos dSmyd4 was restricted to muscle fibres and staining was strongly localised to the cytoplasm (Fig 4C). We used *in situ* hybridisation to confirm whether dSmyd4 expression was restricted to the muscle lineage throughout embryogenesis. dSmyd4 mRNA was expressed throughout the embryonic mesoderm from stage 10 (Fig 4D–K). dSmyd4 was observed in visceral, cardiac and somatic muscle precursors and in late embryogenesis dSmyd4 was strongly expressed in the somatic musculature. This expression pattern indicates that dSmyd4 may play a role in muscle development or function.

### dSmyd4 loss-of-function causes lethality

We used the Gal4-UAS system [26] to induce RNAi against dSmyd4 in different tissues. Two independent insertions of the UAS-RNAi construct were tested, *CG14122R-1* and *CG14122R-3*. In the presence of Gal4 these constructs produce long RNA hairpins that are processed to produce short interfering RNAs. The viability of flies carrying one copy of the UAS-RNAi construct and one copy of a Gal4 driver is summarised in Table 1. Crosses with wild type (*yellow white*; *yw*) flies that do not express Gal4 were used as a negative control. Ubiquitous RNAi using *Act5C*-*Gal4* or *Da*-*Gal4* resulted in severe levels of lethality. To reduce the possibility that an off-target effect was responsible for this phenotype we specifically induced RNAi in all muscle tissue using *24B*-*Gal4* and *Mef2*-*Gal4*. Crosses between these drivers and the stronger UAS-RNAi insertion, *CG14122R-3*, also resulted in lethality. However, inducing RNAi with the neuronal driver *Elav-Gal4* caused no reduction in viability with either UAS-RNAi line.

**Table 1:** Viability of *Drosophila* with induced RNAi (%)

Gal4 driver line	UAS-RNAi line	
	*R1*/*CyO GFP w+*	*R3*/*CyO GFP w+*
*yw*	92	105
*Elav*-*Gal4*	106	101
*Da*-*Gal4*	1	0
*Act5C*-*Gal4*/*TM6b Tb*	10	7
*24B*-*Gal4*	99	5
*dMef2*-*Gal4*	101	76

Percentage viability is calculated as the number of unbalanced adult escapers recovered as a percentage of the number of *CyO* adult escapers from UAS-RNAi*/CyO GFP w+* x *Gal4* crosses. For the UAS-RNAi*/CyO GFP w+* x *Act5C-Gal4/TM6b* cross the percentage is calculated as the number of unbalanced adult escapers recovered as a percentage of half the number of *CyO* adult escapers. Total progeny from each cross was >100.

### Mesodermal knockdown of dSmyd4 causes eclosion failure

**Figure 5:**
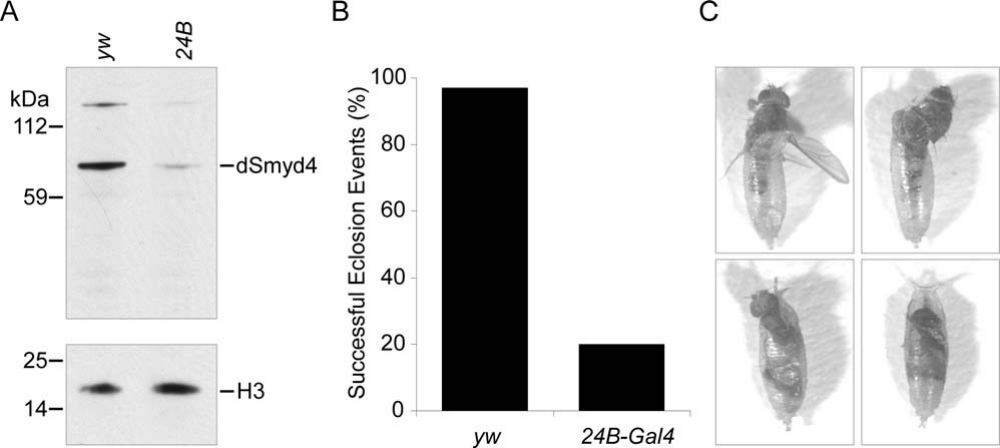
RNAi knockdown of dSmyd4 causes eclosion failure. A, Protein extracts prepared from adult flies were western blotted with anti-dSmyd4 and anti-histone H3 (H3). Flies in which RNAi was induced in the mesoderm (*24B*) from *CG14122R-3* expressed much lower levels of dSmyd4 compared to flies from control crosses (*yw*). B, Flies in which RNAi was induced in the mesoderm (*24B-Gal4*) showed impaired ability to eclose from the pupal case compared to flies from control crosses (*yw*). n = 171 for both crosses. C, Photographs of *CG14122R-3*/*24B-Gal4* flies that remained trapped in the pupal case and died during various stages of eclosion.

We examined the protein level of dSmyd4 when RNAi was induced with the muscle-specific driver *24B*-*Gal4*. Inducing RNAi in the mesoderm was sufficient to knockdown almost all dSmyd4 protein expression in adult flies (Fig 5A). The level of a high molecular weight band recognised by the antibody was also reduced, suggesting that this is a modified form of dSmyd4. When RNAi was induced ubiquitously or in the mesoderm we observed large numbers of dead pupae. These flies had died just prior to, or during, eclosion, the stage when adult flies escape from the pupal case. When RNAi was induced in the mesoderm using *24B*-*Gal4*, fewer than 20% of flies were able to eclose (Fig 5B). Many flies initiated the eclosion process, but became trapped and died partially emerged from the case (Fig 5C). Eclosion requires peristaltic movement of the abdominal muscles to enable flies to escape from the pupal case [27]. Most knockdown flies were able to perform rupture of the pupal case and those flies that escaped far enough also performed normal wing expansion. This phenotype resembled that of temperature sensitive *dMef2* allelic combinations, raised to the non-permissive temperature during larval development [28]. dMef2 is expressed throughout muscle tissue and is required for embryonic muscle development [29], [30]. The majority of flies lacking *dMef2* during adult myogenesis survive until the late pupal stage but fail to eclose fully [28]. The similarities between the eclosion failure phenotype and expression patterns of dMef2 and dSmyd4 suggest that dSmyd4 is also required for correct muscle function during eclosion.

## Discussion

### The *Drosophila* Smyd family

The *Drosophila* genome contains eleven Smyd genes, more than have previously been annotated in the human or mouse genomes. The large number of family members may allow these proteins to assume a repertoire of functions, or ensure redundancy between family members during development. Further analysis of vertebrate genomes may also reveal larger numbers of Smyd proteins than had previously been anticipated. Studies in vertebrates show that individual Smyd proteins control gene expression in order to fulfil varied functions during development. The tissue specific expression patterns of *Drosophila* Smyd family members suggest that these proteins may play equivalent roles in the development of specific tissues in this species.

### Conserved mechanisms of repression and localisation of Smyd proteins

dSmyd4 represses transcription and recruits HDACs in a manner analogous to vertebrate Smyd1 and Smyd2 [9], [16]. This study gives additional insight into the HDAC co-repressors that are involved in repression by dSmyd4. We have shown that dSmyd4 interacts with both dHDAC3 and Ebi, components of the SMRTER co-repressor complex. This contrasts with mammalian Smyd2 protein, which interacts with the Sin3A-HDAC complex [9]. We were unable to detect an interaction between dSmyd4 and HDAC1-containing NuRD, CoREST and Sin3A co-repressors by immunoprecipitation. Nevertheless, a common feature of dSmyd4 and vertebrate Smyd2 and Smyd1 is the association of a potential methyltransferase with histone deacetylase activity in a single complex. This implies that a primary role of these proteins is to co-ordinate changes in modification status at their target sites.

We have described a direct interaction between dSmyd4 and the N-terminal regions of dHDAC1 and dHDAC3. There is a high level of identity between *Drosophila* and vertebrate class I HDACs, especially at the N-termini where this interaction occurs, therefore this interaction may be relevant to recruitment of HDACs by Smyd family members in other species. The recruitment of HDAC co-repressor complexes by MYND domains is also of clinical importance. AML/MTG8 fusions lead to the aberrant recruitment of HDAC co-repressor complexes in the development of leukaemia [13]. The MTG8 MYND domain interacts with components of these complexes, but the interaction between the MYND domain of MTG8 and HDACs is poorly described. The novel interaction described here may also apply to other interactions such as these.

The cytoplasmic over-expression pattern of dSmyd4 resembles that of vertebrate Smyd2 [9], providing another parallel between vertebrate and invertebrate Smyd proteins. However, a more relevant indicator of biological function is the distribution of endogenous protein. We show here that endogenous dSmyd4 is predominantly nuclear in S2 cells. The strong cytoplasmic localisation of dSmyd4 in embryos suggests that in addition to its activity as a transcriptional repressor, dSmyd4 may perform additional cytoplasmic functions, for example the methylation of non-histone substrates. This raises additional parallels with Smyd2, since a cytoplasmic role has been suggested for this protein [6]. The cell-type dependent localisation of endogenous dSmyd4 raises interesting questions about how the localisation of dSmyd4 is regulated. The subcellular localisation of human Smyd3 is regulated in a cell cycle dependent manner and analogous developmental regulation may be required for the function of other Smyd proteins such as dSmyd4 [7].

### Requirement for dSmyd4 in development

Knockdown of dSmyd4 in muscle tissue resulted in reduced rates of survival. dSmyd4 was expressed during embryogenesis, yet the majority of knockdown flies died at the late pupal stage suggesting that there is a greater requirement for dSmyd4 in processes involved in adult myogenesis. This may be due to redundancy between Smyd proteins during embryogenesis since CG8503 and CG18136 are also expressed in muscle tissue at this time [24]. The majority of knockdown flies were not able to escape from the pupal case but performed other eclosion behaviours normally. The neural networks and signalling required for eclosion [27] therefore appear to be intact, indicating that dSmyd4 is likely to play a role in controlling muscle development or function. Identifying the precise nature of the eclosion defect caused by dSmyd4 knockdown will be an important step in understanding the function of this and other Smyd proteins in the development of multicellular organisms. Much is known about the transcription factors involved in *Drosophila* muscle development [29], [30], [31], [32] but little is understood about how chromatin structure is regulated during this process. dSmyd4 is a good candidate to direct chromatin remodelling during muscle development. Smyd1 is required for cardiac development in vertebrates [8], [16] and a number of other *Drosophila* Smyd proteins appear to be specifically expressed in muscle. These results suggest that members of the Smyd family play conserved roles in muscle development in both vertebrate and invertebrate species. *Drosophila* provides a tractable system for the analysis of gene function, for example testing genetic interactions with other genes involved in muscle development. Analysis of mutants in *dSmyd4* and other *Smyd* genes using this approach may also shed light on conserved aspects of Smyd function in vertebrates.

This study presents the first analysis of both Smyd proteins in *Drosophila* and of a Smyd4 homologue. It appears that aspects of mechanism and function are conserved between *Drosophila* and vertebrate Smyd proteins. The repression of transcription by SMRTER complex recruitment and the requirement of dSmyd4 for survival highlight the importance of this protein family as transcriptional modulators of developmental processes.

## Materials and Methods

### Identification of *Drosophila* Smyd homologues

BLAST searches against the *Drosophila* annotated proteins database were performed using each of the human Smyd proteins. Candidate Smyd proteins were analysed for the presence of SET and MYND domains by reference to Uniprot and direct comparison with consensus sequences.

### Cloning

cDNA clones from the Berkeley *Drosophila* Genome Project [33] were obtained from Geneservice (Cambridge, UK): CG11160, RE25548; CG12119, RE62495; CG14122, RE32936; CG14590, AT24727; CG1868, LD26420; CG7759, HL04910; CG8378, LD29892; CG8503, GH11294; dHDAC1, GM14158; dHDAC3, LD23745. Coding regions were amplified by PCR and cloned into the S2 expression vector pRmHa3.

### S2 cell culture

*Drosophila* S2 cells (Drosophila Genomics Resource Centre) were grown at 25°C in Schneider's Medium supplemented with 10% foetal calf serum and antibiotics. S2 cells were split the day before transfection and were plated at a density of 0.5×10^6^ per well of a 24 well plate on the day of transfection. Cells were transfected using FuGENE HD (Roche). Expression from pRmHa3 was induced with 0.7 mM CuSO_4_.

### LexA dependent reporter assay

*Drosophila* S2 cells were transfected with 50 ng pAc5.1 (Invitrogen) encoding dSmyd4 fused to LexA at the C-terminus, or LexA alone. Cells were co-transfected with 100 ng pRLAct5C, encoding *Renilla* luciferase downstream of an *Actin5C* promoter and 500 ng pGL2LexA, encoding firefly luciferase downstream of four interspersed LexA sites/*Drosophila* heat shock elements and a minimal *hsp70* promoter. The constructs were based on pRL and pGL2 respectively (Promega). Cells were harvested two days after transfection. The Dual-Luciferase Reporter system (Promega) was used to measure firefly and *Renilla* luciferase luminscence according to the manufacturer's directions. Each firefly luciferase reading was normalised to its partner *Renilla* luciferase reading to control for cell number/viability and transfection efficiency. Results were the mean of three transfections and the mean value for LexA alone was set to an arbitrary value of one.

### Co-immunoprecipitation

S2 cells expressing dSmyd4-FLAG and dHDAC1-HA or dHDAC3-HA were lysed in IPB250 (20 mM Tris pH 8.0, 250 mM NaCl, 0.4% NP-40, 1 mM DTT, 1 mM EDTA and Roche protease inhibitors). S2 cells expressing dSmyd4-FLAG and dMi2-HA, Ebi-HA, Sin3A-HA or CoREST-HA were lysed in IPB150 (20 mM Tris pH 8.0, 150 mM NaCl, 0.1% NP-40, 1 mM DTT, 1 mM EDTA and Roche protease inhibitors). Cell extracts were incubated with anti-FLAG M2 sepharose (Sigma) for 2 h at 4°C. Sepharose was washed extensively in the lysis buffer and proteins were eluted in 500 ng/µL FLAG peptide (Sigma). Bound proteins were visualised by western blotting with anti-FLAG M2 (1∶5000; Sigma) and anti-HA (1∶2000; Roche).

### HDAC pulldowns

The region encoding the MYND domain fragment of dSmyd4 (amino acids 208–377) was cloned into pGex4T1 (Pharmacia) to generate an N-terminal GST fusion. GST-MYND was purified in phosphate buffered saline (PBS) with 0.1% Triton X-100 on glutathione sepharose. ^35^S-labelled dHDAC1 and dHDAC3 were generated by *in vitro* translation using TNT Quick Coupled Transcription/Translation System (Promega). 10 µg GST-MYND bound to glutathione sepharose was incubated with ^35^S-labelled dHDAC1 or dHDAC3 in PDB (20 mM Tris pH 8.0, 200 mM NaCl, 50 mM KCl, 0.5% Triton X-100, 1 mM DTT, 1 mM EDTA) for 2 h at 4°C. Sepharose was washed extensively with PDB. Bound proteins were separated by SDS PAGE and visualised using a Typhoon scanner (GE Healthcare). dHDAC1-his_6_ and dHDAC3-his_6_ were expressed from pET28 (Novagen) and were purified on Ni-NTA agarose (Qiagen) according to the maufacturer's directions. dHDAC1-his_6_ and dHDAC3-his_6_ were eluted from the agarose by incubation with 250 mM imidazole for 10 min at room temperature. 10 µg GST-MYND bound to glutathione sepharose was incubated with 20 µg dHDAC1-his_6_ or dHDAC3-his_6_ in HisPDB (20 mM Tris pH 8.0, 150 mM NaCl, 50 mM KCl, 0.25% Triton X-100, 1 mM DTT, 1 mM EDTA) for 2 h at 4°C. Proteins were separated by SDS PAGE and visualised by western blotting with anti-polyhis (1∶4000; Sigma).

### Generation of anti-dSmyd4

A polyclonal antibody against dSmyd4 was raised in rabbits using the GST-MYND protein fragment. Immunisation was performed by Eurogentec. GST-MYND was blotted onto nitcrocellulose membrane and incubated with the final bleed diluted in PBS. The membrane was washed extensively in PBS and the antibody was eluted from the membrane in 200 mM glycine pH 2.5 and immediately neutralised. The affinity purified antibody specifically recognised dSmyd4 with low background in western blots (1∶2000) and immunofluorescence.

### Immunofluorescence

S2 cells were fixed in 4% formaldehyde. Embryos were fixed and devitellinised according to Kosman *et al.* [34]. The following antibody dilutions were used for stainings: anti-FLAG M2 (Sigma), 1∶2000; anti-HA 3F10 (Roche), 1∶500; anti-dSmyd4, 1∶250 for S2 cells, 1∶50 for embryos. Alexafluor conjugated secondary antibodies (Molecular Probes) were used at 1∶250. Nuclei were visualised with DAPI (500 ng/mL). Embryos and S2 cells were imaged using confocal microscopy.

### *In situ* hybridisation

Full-length dSmyd4 cDNA was used to generate a digoxygenin-labelled anti-sense probe using the DIG labelling kit (Roche) according to the manufacturer's directions. Embryos were fixed according to Kosman *et al.* [34]. Hybridisation was performed according to the protocols of Kosman *et al.* and Tomancak *et al.* [24], [34] except a 5 min incubation at 90°C was used in place of proteinase K treatment. Hybridised RNA was visualised with anti-digoxygenin-alkaline phosphatase and NBT/BCIP (Roche). Embryos were staged according to Campos Ortega and Hartenstein [35].

### Fly stocks and RNAi

Fly stocks were maintained at 25°C on standard medium. RNAi was induced using the UAS-Gal4 system [26]. *CG14122R-1* and *CG14122R-3* UAS-RNAi lines were obtained from the RNAi fly project, NIG/MITILS, Japan. UAS-RNAi stocks balanced with *CyO GFP w+* were crossed to a variety of Gal4 expressing drivers: *Act5C-Gal4* (Y. Hiromi, Bloomington Stock centre); *Da-Gal4* and *24B-Gal4* (gifts from M. Bienz); *Elav-Gal4*; *Mef2-Gal4* (a gift from S. Huelsman). Viability of flies containing one copy of the UAS-RNAi insertion and one copy of a Gal4 driver was compared to that of *CyO* flies from the same cross. To analyse the eclosion defect non-GFP larvae from the crosses *CG14122R-3/CyO GFP w+* x *24B-Gal4* and *CG14122R-3/CyO GFP w+* x *yw* were selected and allowed to develop.

## Supporting Information

Table S1 Domain annotation of *Drosophila* Smyd homologues
(0.06 MB DOC)

Figure S1 Alignments of *Drosophila* Smyd proteins and human Smyd proteins. A, SET domain. B, MYND domain. The two MYND domains of CG8503 are denoted zf1 and zf2 respectively. In A and B identical residues are shaded black and similar residues are shaded grey.
(1.21 MB TIF)

Figure S2 Subcellular localisation of *Drosophila* Smyd proteins HA-tagged *Drosophila* Smyd proteins were over-expressed in S2 cells and visualised by immunofluorescence using anti-HA. Nuclei were visualised with DAPI.
(8.33 MB TIF)

Figure S3 Anti-dSmyd4 specifically recognises dSmyd4 in western blots and immunofluorescence. A, dSmyd4-HA was over-expressed in S2 cells and visualised by immunofluorescence using anti-HA and anti-dSmyd4. Nuclei were visualised with DAPI. B, Protein extracts were western blotted with anti-dSmyd4, pre-immune serum or anti-FLAG. S2 + FLAG, S2 cell extract with over-expressed dSmyd4-FLAG; S2, S2 cell extract; FLAG IP, immunoprecipitated dSmyd4-FLAG.
(3.72 MB TIF)
