# Supplementary material for: A Drosophila Smyd4 Homologue Is a Muscle-Specific Transcriptional Modulator Involved in Development
Source: PLoS One. 2008 Aug 20;3(8):e3008. doi: 10.1371/journal.pone.0003008 (PMC2500188; doi:10.1371/journal.pone.0003008)
Supplement: Table S1 — Domain annotation of Drosophila Smyd homologues (0.06 MB DOC) [file pone.0003008.s001.doc]

TABLE S1

**Domain annotation of *Drosophila* Smyd homologues**

|  | **Additional domains annotated in UniProt** | **Subcellular over-expression pattern** | **Adult tissues with enriched expression:**  **Fly Atlas** | **Embryonic localisation:**  **BDGP *in situ* hybridisation** |
| --- | --- | --- | --- | --- |
| **CG18136** |  | ND | Mesoderm | Somatic muscle |
| **MSTA** |  | ND | Mesoderm; head | ND |
| **CG8503** | Isopenicillin *N* synthase domain; two MYND domains | Cytoplasmic | Mesoderm; ovary | Throughout mesoderm |
| **CG12119** |  | Predominantly nuclear | Male accessory glands | ND |
| **CG14590** |  | ND | ND | ND |
| **CG9642** |  | ND | Not specific | ND |
| **CG11160** |  | Predominantly cytoplasmic | Mesoderm | No staining |
| **CG8378** |  | Predominantly cytoplasmic | Ovary; testis; male accessory glands | ND |
| **CG7759** | Tetratricopeptide repeats | Cytoplasmic aggregates | Mesoderm; testis | ND |
| **CG14122** | Tetratricopeptide repeats | Predominantly cytoplasmic | Mesoderm | Throughout mesoderm |
| **CG1868** |  | Predominantly cytoplasmic | Ovary; testis | Early embryo |
