# Supplementary figures and images for: A Drosophila Smyd4 Homologue Is a Muscle-Specific Transcriptional Modulator Involved in Development
Source: PLoS One. 2008 Aug 20;3(8):e3008. doi: 10.1371/journal.pone.0003008 (PMC2500188; doi:10.1371/journal.pone.0003008)

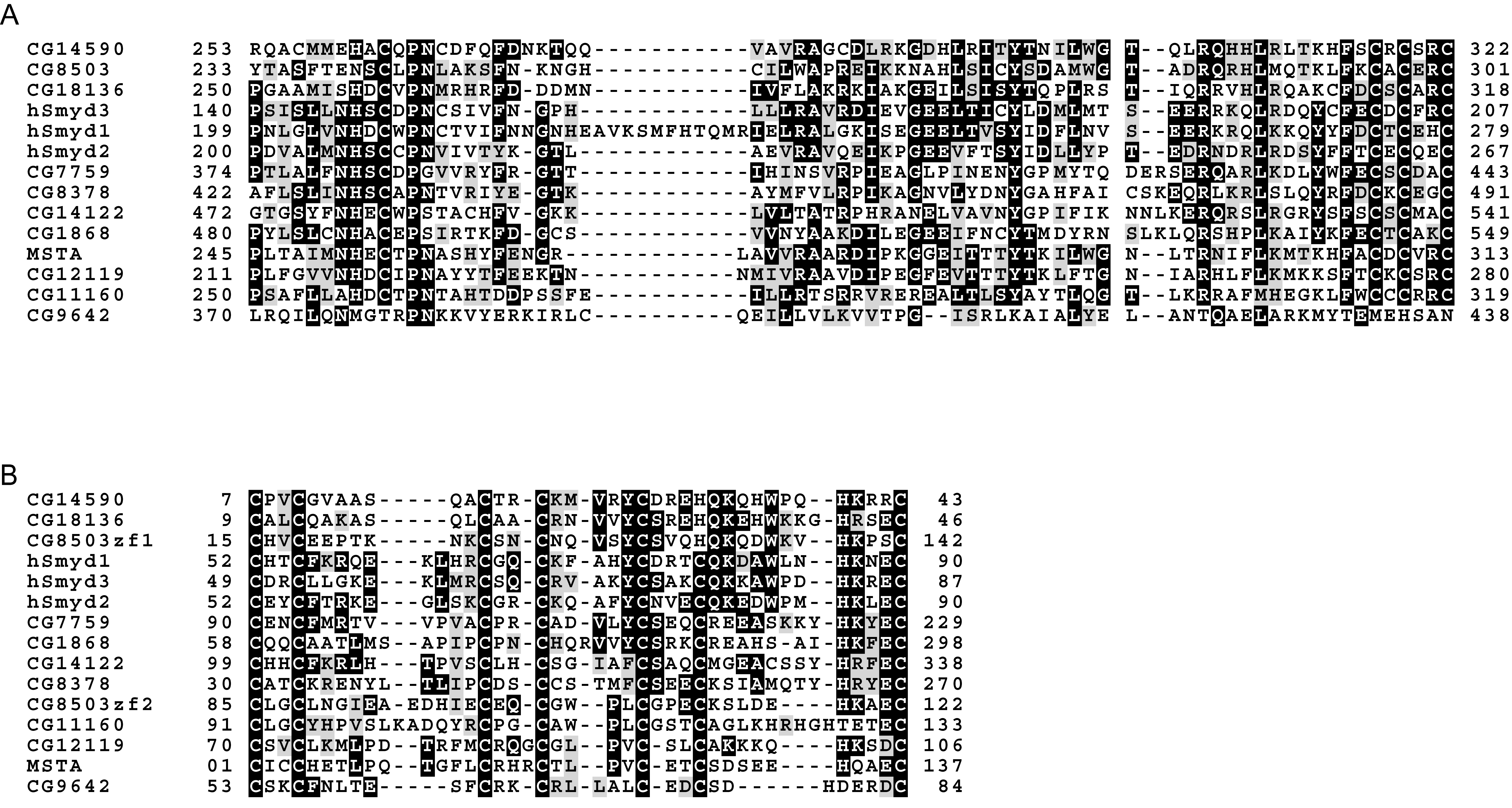

Supplement: Figure S1 — Alignments of Drosophila Smyd proteins and human Smyd proteins. A, SET domain. B, MYND domain. The two MYND domains of CG8503 are denoted zf1 and zf2 respectively. In A and B identical residues are shaded black and similar residues are shaded grey. (1.21 MB TIF) [file pone.0003008.s002.tif]

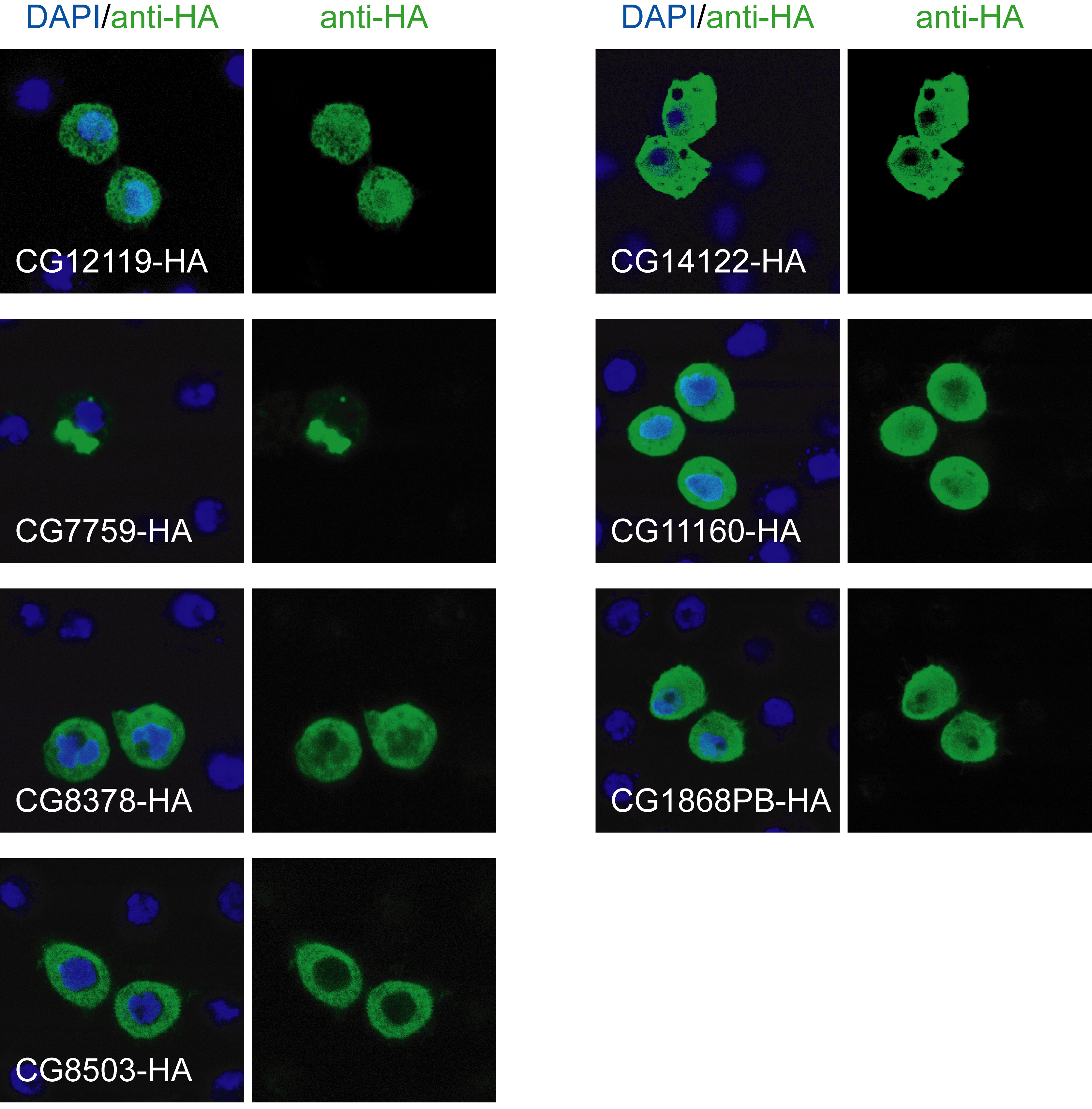

Supplement: Figure S2 — Subcellular localisation of Drosophila Smyd proteins HA-tagged Drosophila Smyd proteins were over-expressed in S2 cells and visualised by immunofluorescence using anti-HA. Nuclei were visualised with DAPI. (8.33 MB TIF) [file pone.0003008.s003.tif]

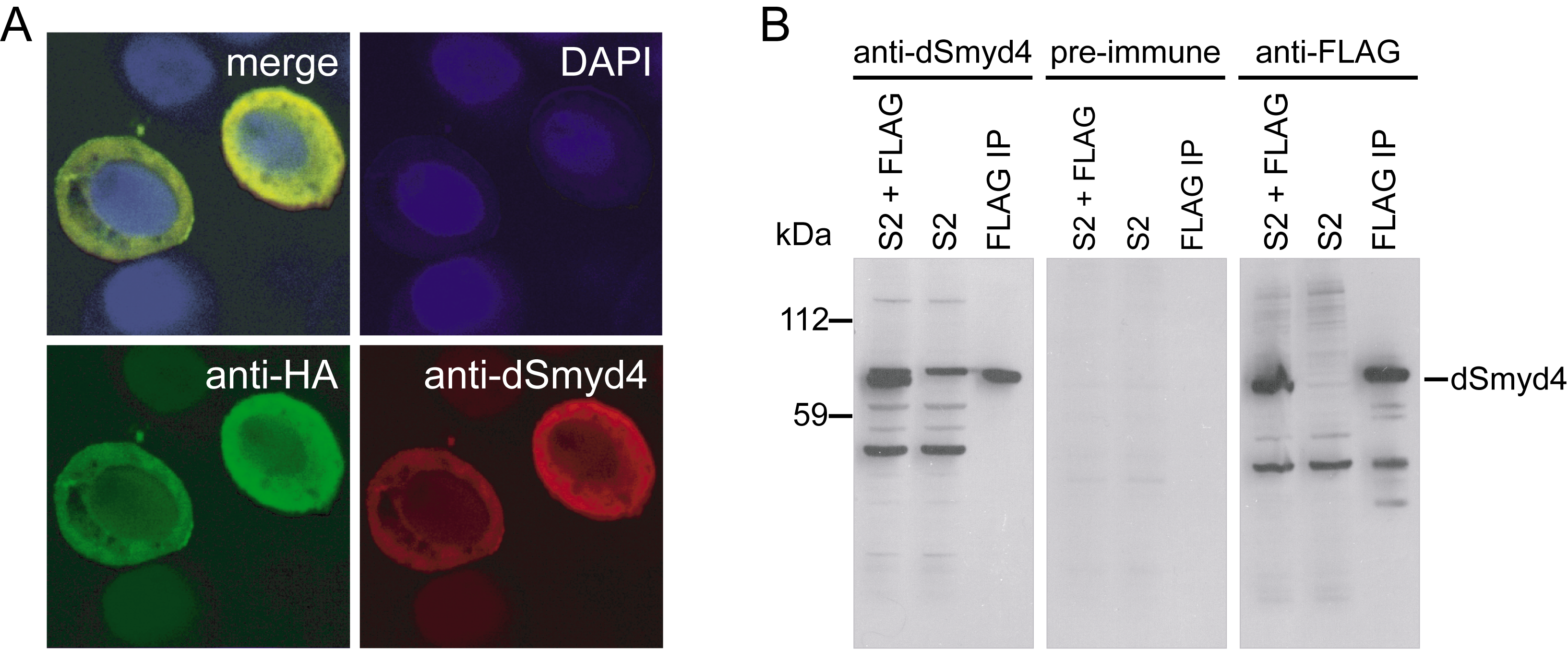

Supplement: Figure S3 — Anti-dSmyd4 specifically recognises dSmyd4 in western blots and immunofluorescence. A, dSmyd4-HA was over-expressed in S2 cells and visualised by immunofluorescence using anti-HA and anti-dSmyd4. Nuclei were visualised with DAPI. B, Protein extracts were western blotted with anti-dSmyd4, pre-immune serum or anti-FLAG. S2 + FLAG, S2 cell extract with over-expressed dSmyd4-FLAG; S2, S2 cell extract; FLAG IP, immunoprecipitated dSmyd4-FLAG. (3.72 MB TIF) [file pone.0003008.s004.tif]
